# Impact of Vaccination Rates and Gross Domestic Product on COVID-19 Pandemic Mortality Across United States

**DOI:** 10.1101/2024.01.21.24301582

**Published:** 2024-01-22

**Authors:** Olga Matveeva, Aleksey Y. Ogurtsov, Svetlana A. Shabalina

**Affiliations:** 1 Sendai Viralytics LLC, Acton, MA 01720, USA; 2 National Center for Biotechnology Information, National Library of Medicine, National Institutes of Health, Bethesda, MD 20894-6075, USA

**Keywords:** Vaccination Rate, Pandemic Excess Mortality, COVID-19, Gross Domestic Product

## Abstract

**Objective:**

To investigate the relationship between vaccination rates and excess mortality during distinct waves of SARS-CoV-2 variant-specific infections, while considering a state’s GDP per capita.

**Methods:**

We ranked U.S. states by vaccination rates and GDP and employed the CDC’s excess mortality model for regression and odds ratio analysis.

**Results:**

Regression analysis reveals that both vaccination and GDP are significant factors related to mortality when considering the entire U.S. population. Notably, in wealthier states (with GDP above $65,000), excess mortality is primarily driven by slow vaccination rates, while in less affluent states, low GDP plays a major role. Odds ratio analysis demonstrates an almost twofold increase in mortality linked to the Delta and Omicron BA.1 virus variants in states with the slowest vaccination rates compared to those with the fastest (OR 1.8, 95% CI 1.7-1.9, p < 0.01). However, this gap disappeared in the post-Omicron BA.1 period.

**Conclusion:**

The interplay between slow vaccination and low GDP per capita drives high mortality.

## Graphical abstract

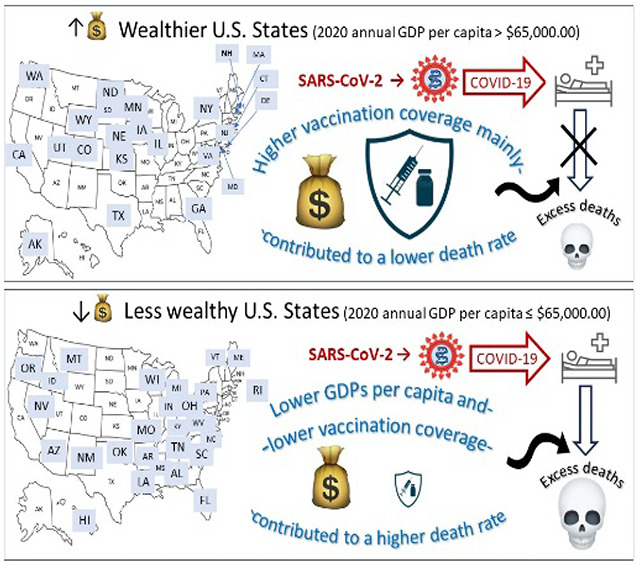

## Introduction

In May 2023, the U.S. Centers for Disease Control and Prevention (CDC) and the World Health Organization (WHO) officially declared the end of the COVID-19 pandemic as a public health emergency. However, despite the decline in COVID-19 infections, persistent morbidity, and mortality, coupled with the looming threat of future significant outbreaks, necessitate a thorough examination of the effectiveness of past pandemic control strategies. The considerable variations in mortality rates among U.S. states underscore the pressure for a meticulous analysis and strategic planning for future public health challenges.

We chose to assess excess mortality because it provides a more comprehensive measure of the overall impact of the pandemic than reported COVID-19 deaths alone. It accounts for both direct COVID-19 fatalities and indirect consequences, such as disruptions in healthcare systems, delayed medical treatments, and the socioeconomic fallout.

While existing research has predominantly focused on individual factors such as vaccination rates ^1, 2, 3, 4, 5, 6, 7^ or socioeconomic parameters ^7, 8, 9^, associated with reduced pandemic excess mortality or COVID-19 fatalities, this study takes an approach by simultaneously considering both vaccination rates and a state’s economic well-being, measured by its annual GDP per capita. Crucially, a state’s GDP per capita serves as an indicator of its economic capacity. States with lower GDP per capita may face greater challenges related to healthcare infrastructure, resource accessibility, and socioeconomic support systems, potentially contributing to elevated rates of pandemic all-cause mortality.

Previous studies ^10, 11, 12^ have explored the impact of various factors on pandemic excess mortality, yet the specific relationships within the U.S. have not been extensively investigated.

The statistical analysis conducted in this study focused on examining the relationship between the three variables and specifically focused on the 50 states of the United States. By integrating both vaccination rates and economic indicators, this research seeks to shed light on the intricate dynamics influencing excess mortality, paving the way for more effective and targeted public health interventions.

## Methods

### Excess mortality data and CDC models

Data on excess mortality in the United States were sourced from the US Centers for Disease Control and Prevention (CDC) ^13^. We downloaded the file “National and State Estimates of Excess Deaths” for our analysis. To ensure proper comparisons, we normalized the absolute values of excess mortality for each specific period using the population size of each state obtained from the United States Census Bureau’s 2020 data ^14^. For this normalization process, we used the file “Annual Estimates of the Resident Population for the United States, Regions, States, District of Columbia, and Puerto Rico: April 1, 2020, to July 1, 2022.”

CDC’s method for calculating excess deaths involves a modeling approach that compares recent mortality data to historical trends dating back to 2013 ^15^. This analysis aims to identify significant increases in all-cause mortality.

### Vaccination dataset

Vaccination data for the United States was also procured from the US Centers for Disease Control and Prevention (CDC) ^16^. We downloaded the file “COVID-19 Vaccinations in the United States, Jurisdiction” and extracted data that were relevant to the dates October 2, 2021, and January 2, 2022, from the column “titled “Series_Complete_Pop_Pct” and booster doses from the column titled “Additional_Doses_Vax_Pct”. Information about the file is available in CDC ^16^. Additionally, we included a dataset on the state’s annual per capita gross domestic product (GDP) per capita for 2020. We obtained this dataset from the Bureau of Economic Analysis ^17^, and used the file “Annual GDP by State and Industry”.

### Statistical analysis

The time intervals for analysis were classified as follows:

- Pre-Delta (February 2020-June 2021): This period represents the initial phase of the pandemic before the emergence of the Delta variant.
- Delta (July 2021-December 2022): The Delta variant was predominant, and mass vaccination was already underway. The Delta-Omicron transition happened in December/January 2022.
- Omicron BA.1 (February 2022-March 2022): The specific period corresponds to prevalence of the first Omicron variant.
- Post-Omicron BA.1 (April 2022-April 2023): This interval covered the period after the emergence of the first Omicron variant. Summary information for statistical analysis from the specified databases for the indicated time intervals was presented in Supplementary file 1 (eTable 1).

All states were categorized based on real GDP per-capita in 2020 using the threshold that was chosen arbitrarily. States with GDP per-capita above $65,000 were classified as higher-income, while those below $65,000 were considered less affluent.

Statistical analysis, including Pearson correlation analysis, linear regression fitting, and Odds Ratio calculations, was performed using Excel functions. In addition, we validated the results using the online tool ^18^.

## Results

Here, we presented a detailed analysis of excess mortality, GDP, and vaccination rates and examined their interplay.

### Visualizing mortality waves and vaccination progress during the pandemic

**Figure 1.**
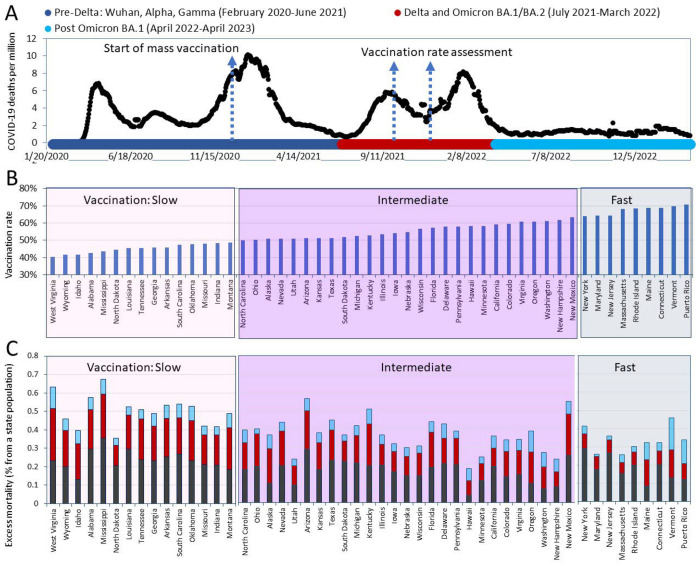
Visualization of Pandemic Mortality Waves/Periods and Vaccination Rates. A. A timeline of daily diagnosed COVID-19 deaths was created using the OWID database and presented along with a scheme of the dominant virus variants causing infections. B. States were categorized into three groups based on their vaccination coverage in ascending order by October 2021. The “fastest” category (light blue background) consists of states that had achieved a vaccination rate of 60% for residents by October 2021 and 70% by January 2022. The “slowest” category (light pink background) includes states in which more than half of the residents remained unvaccinated as of October 2021. The remaining states are placed in the intermediate category (with a purple background). All states and Puerto Rico are shown in the order of the increasing percentage of the state population that had received the primary series of the vaccine as of October 2, 2021. C. Excess pandemic mortality relative to the state’s population in different pandemic periods.

The relationship between mortality dynamics and the COVID-19 pandemic becomes apparent when considering the successive waves of SARS-CoV-2 infections, marked by the emergence and dominance of distinct viral variants. Recorded fatalities can be attributed to both the direct consequences of COVID-19 pathogenesis and its broader impact. The interplay between the evolving viral landscape and host responses elucidates temporal patterns in morbidity and mortality observed across diverse phases of infection waves. Figure 1A provides a visual representation of the dynamics of daily COVID-19 reported deaths throughout the entire pandemic, illustrating distinct waves and periods of infection with SARS-CoV-2 virus variants. Supplementary Figure 1 delineates the chronological order of appearance and dominance of these variants.

As a first step toward comprehending pandemic disease dynamics and evaluating the effectiveness of vaccination efforts, all-cause mortality (excess mortality) was calculated separately for the pre-Delta, Delta-Omicron BA.1, and post-Omicron BA.1 periods (Supplementary file, eTable 1). These periods are marked with different colors in Figure 1A. In the next step of our analysis, we ranked states according to their vaccination rates and divided them into three categories, as shown in Figure 1B. The “fastest” category included states in which the vaccination coverage for residents reached 60% by October 2, 2021, and 70% by January 2, 2022. This category includes the states of Vermont, Connecticut, Maine, Rhode Island, Massachusetts, New Jersey, Maryland, and New York. The “slowest” category includes states where more than half of the residents remained unvaccinated as of October 2, 2021. The states in this category are North Carolina, Montana, Indiana, Missouri, Oklahoma, South Carolina, Arkansas, Georgia, Louisiana, Tennessee, North Dakota, Mississippi, Alabama, Idaho, Wyoming, and West Virginia. The remaining states were categorized as having intermediate vaccination rates.

Finally, we integrated information pertaining to mortality and vaccination rates by calculating and illustrating the number of fatalities in each state based on its respective category and time intervals (Figure 1C). However, the resulting illustration, although showing trends that states with faster vaccination had lower mortality rates, does not allow us to assess these trends quantitatively. Such an assessment was our next goal, and how we went about it is described in the next sections.

### The contrast in excess mortality between different categories of states is most pronounced during the Delta-Omicron BA.1 period

Having defined time intervals and state categories, we compared the distributions of excess mortality in each state category for each interval of interest. In the first pre-Delta period (time interval I - pre-Delta), mass vaccination likely did not play a significant role in preventing excess mortality because it had just begun. In the second period combining Delta and Omicron BA.1 infection waves (time interval II - July 2021 to March 2022), vaccination rates may have played a significant role as vaccination became widespread. In the third period, after the Omicron BA.1 wave (time interval III), the bulk of the population acquired immunity because of direct COVID-19 infection, vaccination, or a combination of both (hybrid immunity). We therefore expect that the association between high vaccination rates and low mortality, if it existed before, may become weak or disappear altogether.

**Figure 2.**
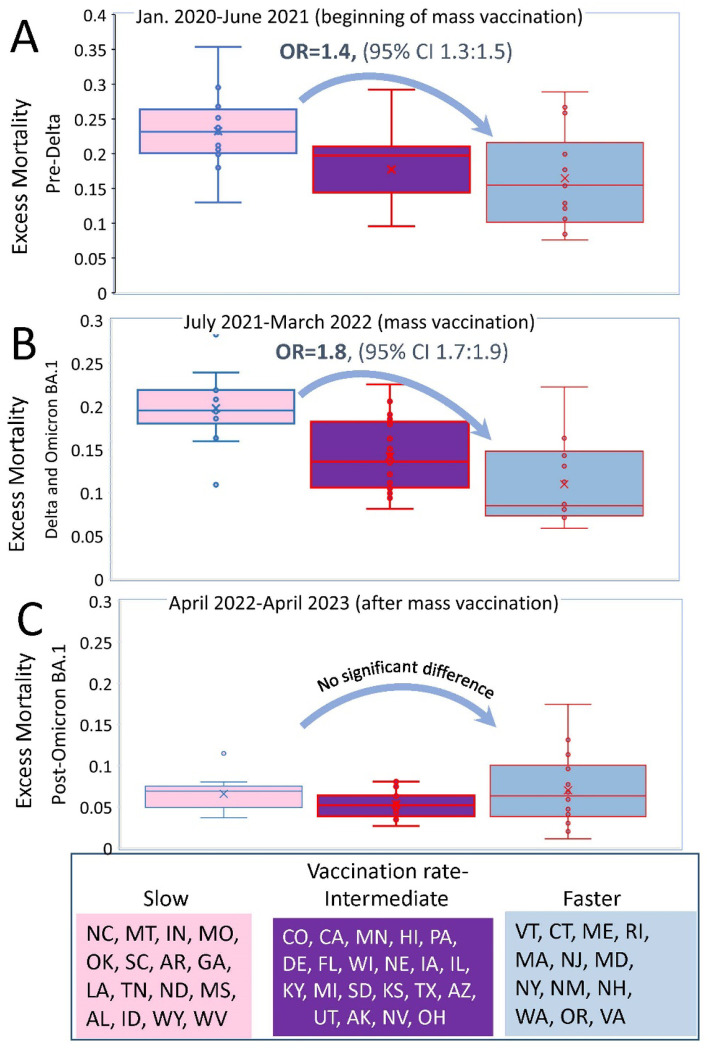
Boxplots Representing Relationships Between Excess Mortality and Vaccination Rates During Different Pandemic Periods. States were categorized in the same manner as described in the legend of Figure 1, and each state’s excess mortality estimate was plotted withing each category: A. For the pre-Delta period, which corresponds to the initial phase of vaccination. B. For the Delta and Omicron BA.1 period, representing an advanced stage of vaccination. C. For the post-Omicron BA.1 period.

Do our expectations align with the observed outcomes? We employed odds ratio analysis to assess mortality, comparing the likelihood of death in one category of states to another. This statistical measure enables us to quantify the association between vaccination rates and mortality. The analysis, conducted over distinct time frames, aligns with our predictions, and is illustrated in Figure 2.

Initially, during the pre-Delta period, distinctions between states with the slowest and fastest vaccination rates were not high (OR=1.41, 95% CI 1.32–1.5, p<0.01). Boxplots in Figure 2A illustrate this contrast for state categories created according to vaccination rates. However, as the Delta period unfolded alongside widespread mass vaccination efforts, the disparities between states with the slowest and fastest vaccination rates became more prominent (OR=1.8, 95% CI 1.66–1.92, p<0.01), as demonstrated in Figure 2B. Importantly, in the post-Omicron BA.1 period, when a significant portion of the population across all states likely attained seroconversion through a combination of vaccination and prior COVID-19 infection, no substantial differences in excess mortality were observed between state categories (Figure 2C).

Low mortality may be attributed not only to vaccination efforts but also to the implementation of various additional measures aimed at minimizing the effects of the pandemic. These may include robust public health initiatives such as mask mandates, social distancing, travel restrictions, quality healthcare, and other measures. The ability to apply these measures may depend on the state’s revenue. To find out how the economic capacity of a state affected excess mortality, we conducted the analyses described in the next section.

### Exploring excess mortality, GDP, and vaccination rates by state category

To understand how a state’s economic status influences its ability to vaccinate quickly and minimize pandemic deaths we analyzed the data using both single-factor and multi-factor analyses. We first examined the simple individual relationships between GDP, vaccination rate, and pandemic deaths using a single-factor analysis (univariable regressions). The insights gained from the single-factor analysis provided a foundation for our subsequent exploration of how these factors might interact in more complex models through two-factor analysis (multivariable regressions).

#### Univariable regression models (vaccination versus GDP per capita)

While investigating the interplay between excess mortality, vaccination rates, and GDP, we identified a distinct relationship between vaccination rates and state economic status. Our analysis revealed a significant positive correlation between these two factors, indicating that, unsurprisingly, states with higher GDP per capita generally achieved faster vaccination rates. This correlation was more pronounced for less wealthy states, suggesting that economic resources play a crucial role in facilitating successful vaccination campaigns in these states. We introduced an arbitrarily chosen threshold of $65,000 to categorize states as more or less wealthy. Specifically, the Pearson correlation between vaccination rates and GDP per capita for the subset of 18 states with incomes below the chosen wealth threshold was R^2^=0.34, p=0.01. However, this correlation is much weaker but still significant for the remaining 32 states (R^2^=0.12, p=0.05). The results of the correlation analysis are presented in more detail in Supplementary file 1 (eTable 2).

Thus, regression analysis reveals that both states’ vaccination rate and economic status are correlated with each other, but the correlation is stronger in a subset of less affluent states. We will delve deeper into this relationship and its implications for excess mortality in the following sections, examining how both state income and vaccination rates individually and synergistically contribute to mortality patterns across the U.S.

#### Univariable regression models (mortality versus GDP per capita, mortality versus vaccination rate)

The next question pertained to the relationship between excess mortality and GDP, as well as between excess mortality and vaccination rate. We aimed to understand whether relevant correlations exist and what form they take. To address these questions, we tested the correlation between excess mortality measured over the combined Delta and Omicron BA.1 pandemic infection waves and the two factors such as annual GDP per capita and vaccination rates, separately using univariable regression fitting.

Notably, regression outputs for the population of all 50 states revealed a highly significant correlation of mortality with both variables separately (R^2^ = 0.48, p <0.01 for GDP; R^2^ = 0.44, p < 0.01 for vaccination rates). Thus, fewer excess deaths were observed in both i) states with higher annual incomes, as well as ii) states with higher vaccination rates.

**Figure 3.**
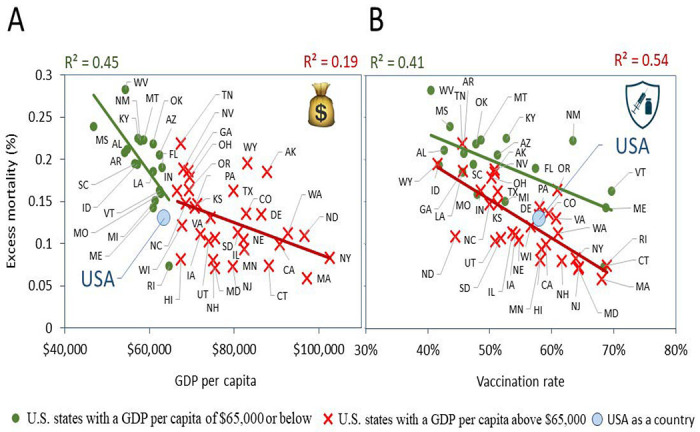
Correlation Analysis of Pandemic Excess Mortality Excess mortality was calculated as the percentage of normalized excess deaths for the state population between July 1, 2021 and March 31, 2022. This timeframe spans the periods of dominance for the Delta and the Omicron BA.1 virus variants. Pearson correlation coefficients reflecting significant relationships (p < 0.05) are shown above the scatter plots. The dependent variable is excess mortality, and the independent variables used were: A. Real GDP per capita for the year 2020. B. Vaccination rate, represented as the percentage of the population fully vaccinated with the primary vaccine series as of October 2.

Correlations diverge across income categories. In a subset of less wealthy states, excess mortality exhibits a stronger negative correlation with state income, while in relatively affluent states (GDP above $65,000), the negative correlation with vaccination rates is more prominent (Compare Figure 3A and Figure 3B).

Our analysis further delved into the trends previously identified. We evaluated the impact of state GDP and vaccination rates on excess mortality, considering separately the Delta wave of infections and the Omicron BA.1 wave. The results consistently revealed similar patterns as in Figure 3 for all income categories and for both infection waves (as depicted in the Supplementary Figure 2). Further detailed analysis shows that lower income states experience higher death rates during both the Delta (Supplementary Figure 2 A) and Omicron BA. 1 waves (Supplementary Figure 2 C), even when their vaccination rates are like more affluent states (Supplementary Figure 2 B and Supplementary Figure D). Of particular interest are the distinctive trends observed in states with incomes exceeding $65,000, where excess mortality showed weak correlation with state GDP but a strong relationship with vaccination rates. Indeed, in these states we observe a significant inverse correlation between vaccination rates and excess mortality. The higher the vaccination rate, the smaller the excess mortality. These distinctive trends are evident for both the Delta wave (Supplementary Figure 1 B) and the Omicron BA.1 wave (Supplementary Figure 2 D).

An inverse correlation between excess mortality and booster administration rate was observed only in wealthy states for the Omicron BA.1 wave (Supplementary Figure 2 E). No significant correlation was detected in lower income states. The lack of correlation in this subset is likely due to inconsistencies in reporting deaths in some states during the very short time interval indicated.

In summary, our analysis reveals the critical roles of both GDP and vaccination rates in shaping excess mortality, with their relative importance varying across income levels. In less wealthy states, excess mortality exhibited a stronger negative correlation with state income, while more affluent states showed a pronounced negative correlation with vaccination rates. This pattern was observed in both the Delta and the Omicron BA.1 infection waves. Moving beyond individual relationships, we proceeded to multivariable regressions to comprehensively understand how the economic status of a state interacts with its vaccination rates in influencing pandemic associated deaths.

#### Multivariable regression models

Upon discovering the correlation between vaccination rates and state economic status and recognizing the correlation of both variables with excess mortality, we faced the challenge of determining whether each of these factors could independently contribute to the estimation of excess mortality. If so, does the weight of their contribution depend on whether the state is wealthier or less wealthy? To address these questions, we created three models: one considering all states and two analyzing subsets created based on state economic capacity (GDP). This strategy enabled us to assess the influence of the two input variables in more affluent and less wealthy state subsets separately.

**Figure 4.**
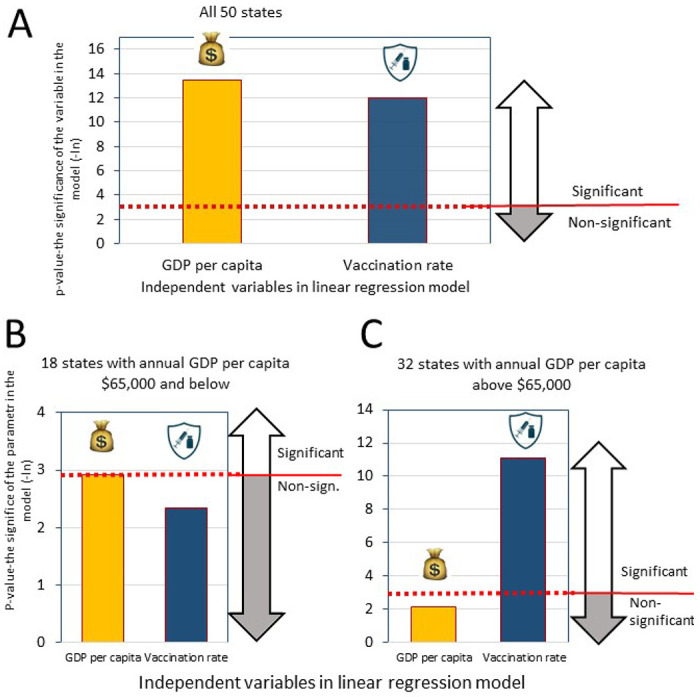
Significance Levels in Multivariable Regression Models Predicting Excess Mortality Considering Different Subsets of States Based on GDP. The significance levels of input parameters in linear regression models used to predict excess mortality associated with the pandemic are depicted. Mortality serves as the regression dependent variable, while GDP per capita and vaccine immunization rates are the independent variables. Each column illustrates the significance level (in negative natural logarithmic values) of a respective input parameter. The models cover the pandemic duration, spanning from Delta variant dominance to the Omicron BA.1 periods (July 1, 2021, to March 31, 2022). The red horizontal line indicates the significance level of p=0.05. The models were created for: A. All 50 states B. States with a GDP above $65,000 C. States with a GDP below $65,000

In the output of the regression model, which involved the analysis of all states, both independent variables were identified as highly significant contributors affecting excess mortality (Figure 4A). However, the further exploration of state subsets classified by GDP revealed intriguing trends. In states with incomes below $65,000 low GDP played a more pronounced and statistically significant role in predicting high mortality (Figure 4B). In contrast, for states with GDP exceeding $65,000, the contribution of GDP in the regression model did not reach statistical significance, and only the vaccination rate represents a statistically significant variable (Figure 4C). Detailed results of the multivariable regression analysis are presented in Supplementary file 1 (eTable 3).

In summary, results obtained from univariable, and multivariable regression analysis are consistent with each other. We reveal a cooperative effect of GDP and vaccination rates on mortality, while acknowledging their intercorrelation. Both factors influence mortality, but their relative importance varies across state wealth categories. In wealthier states (above $65,000 GDP per capita), slow vaccination rates become the dominant driver of excess mortality, outweighing the impact of lower GDP. Conversely, poorer states experience higher mortality even with comparable vaccination rates to wealthier states.

## Discussion

Our current study of US states revealed a similar pattern observed in our previous analysis of European countries ^11^. Interestingly, the relative importance of GDP and vaccination rates in predicting excess mortality shifted with economic context. In countries or states with lower income levels, GDP played a more prominent role, while wealthier countries or states exhibited a stronger association with vaccination rates.

Why do countries or states with lower economic capacity demonstrate higher pandemic-associated fatalities, even if they have similarly high vaccination coverage as wealthier countries or states? A plausible explanation for this phenomenon is that states with lower GDPs, compared to those with higher GDPs, had fewer effective measures to complement vaccination in preventing COVID-19 infections. The deficiency in these measures likely contributed to the higher mortality rates observed in these states. In contrast, wealthier countries or states have enough resources to implement pandemic mitigation strategies and depend mainly on vaccination rates.

The greatest impact of vaccination rates in minimizing excess mortality was observed during periods dominated by the Delta and Omicron BA. 1 variants. This suggests that, despite the vaccines being designed to elicit immunity against the S-protein of the original Wuhan virus variant, they continued to be effective in safeguarding against viral variants that underwent significant antigenic drift, particularly in the S-protein.

In the period following the dominance of Omicron BA.1, most likely a significant proportion of the population in all states had already achieved immunity, which in states with low vaccination rates was offset by high rates of natural infections. These infections resulted in excess deaths, but also contributed to a strengthening of population immunity and this strengthening may have been substantial because of the very large number of infections that had occurred by the end of March 2022 in most states. Highly likely that nearly all states had achieved a comparable level of immunity by that point.

Our analysis demonstrates a progressively stronger association between low vaccination rates and high excess mortality that peaked during the Delta period. Eventually, death rates converge across states in the post-Omicron BA.1 period, most likely due to widespread seroconversion from natural infections.

The findings from our analysis of both European countries and U.S. states offer insights for shaping strategies in future pandemics or infections surges of a lesser degree. Understanding the patterns observed can inform proactive measures to mitigate the impact, particularly in regions with lower economic capacities.

### Study limitations

#### Dependence on CDC Data:

Our study relies heavily on state vaccination and mortality data provided by the CDC. The absence of an independent data source introduces a limitation, as we are unable to verify or account for potential measurement errors.

#### Excess mortality model estimations:

The accuracy of our study is contingent upon the excess mortality model estimations developed by CDC staff.

### Practical implications

#### Addressing socioeconomic disparities:

Our study consistently identifies a correlation between lower economic capacity and higher pandemic-associated fatalities, even when vaccination coverage is similar. Recognizing the influence of income levels, specifically GDP per capita, on pandemic excess mortality highlights the critical need to address socioeconomic disparities in public health initiatives. Additional resources are crucial for states with lower economic capacities to bolster pandemic mitigation strategies.

#### Resource allocation and addressing vaccine hesitancy:

Public health resources and efforts can be strategically allocated based on the economic context of each state. In wealthier states, where pandemic mitigation strategies are more feasible, the emphasis shifts to vaccination rates. Additional resources are needed to enhance vaccination infrastructure and address vaccine hesitancy in all states, excluding those considered vaccination front-runners.

#### Vaccine research and new platforms:

While our data highlight the essential role of vaccination in reducing pandemic mortality before the Omicron era, its effectiveness in preventing deaths did wane over time. This decline might be partly attributed to widespread natural COVID-19 infections, potentially boosting population immunity to levels like or exceeding those achieved through vaccination. Despite this, subsequent infection waves continued, tragically leading to further COVID-19 deaths. The development of broad-spectrum COVID-19 vaccines that target multiple variants, induce mucosal immunity, and offer durable protection remains a crucial public health need.

## Conclusions

In states with higher incomes (above $65,000), vaccination rates played a crucial role in reducing mortality, while GDP had a less pronounced and statistically insignificant impact. In contrast, in less affluent states, state income had a significant and pronounced impact. This pattern holds true for both the Delta and Omicron BA.1 infectious waves.

Our data demonstrate that vaccination played a crucial role in reducing population pandemic mortality up until the post-Omicron BA.1 era. Its role became less visible in this era, likely due to widespread natural COVID-19 infections contributing to population immunity at a comparable level to vaccines or even higher.

The category of front-runner states with the fastest vaccination campaigns that other states can model includes Vermont, Connecticut, Maine, Rhode Island, Massachusetts, New Jersey, Maryland, and New York.

## Supplementary Material

Supplement 1

## Data Availability

Our work was performed with publicly available datasets. All data generated or analyzed during this study is provided as supplementary information files.
