# Supplementary material for: Impact of Vaccination Rates, Pre-Pandemic Life Expectancy, Economic Status and Age on COVID-19 Excess Mortality Across United States
Source: medRxiv. 2024 Aug 12:2024.01.21.24301582. Originally published 2024 Jan 22. Preprint. [Version 2] doi: 10.1101/2024.01.21.24301582 (PMC10836123; doi:10.1101/2024.01.21.24301582)

medRxiv preprint doi: https://doi.org/10.1101/2024.01.21.24301582; this version posted August 12, 2024. The copyright holder for this preprint (which was not certified by peer review) is the author/funder, who has granted medRxiv a license to display the preprint in perpetuity.
It is made available under a CC-BY 4.0 International license .

Supplementary Figure 1. Relationships between state-specific pandemic mortality, and GDP per capita Excess mortality was calculated as the percentage of normalized excess deaths for the state population from January 1, 2020, to July 31, 2022. The Pearson correlation coefficients displayed above the scatterplots indicate statistically significant correlations ($p < 0.05$). (A) Relationship of age-unadjusted pandemic mortality vs. GDP (B) Relationship of age-adjusted pandemic mortality vs. GDP. of age-unadjusted vs. age-adjusted state specific mortalities.

Supplementary Figure 2. Chronological Emergence of SARS-CoV-2 Dominant Variants in the USA

Supplementary Figure 3. Correlation analysis of pandemic excess mortality for Delta and Omicron BA.1 periods separatly Only statistically significant correlations ($p < 0.05$) are presented above the corresponding scatter plots. Excess mortality that was not adjusted for age is depicted for: (A) Delta period in relation to the annual actual GDP per capita in 2020. (B) Delta period in relation to vaccination rates, expressed as a percentage of the state's fully vaccinated population as of October 2, 2021. (C) Omicron BA.1 period in relation to GDP per capita. (D) Omicron BA.1 period in relation to vaccination rates, calculated as a percentage of the state's fully vaccinated population as of January 2, 2022. (E) Omicron BA.1 period in relation to booster administration rates, calculated as a percentage of the state's population that received an additional dose as part of the primary vaccine series as of January 2, 2022.

Supplementary Figure 4. Comparison of relationships of vaccination coverages with pandemic excess mortality estimated with and without adjustement for age Both estimates were perforemd for the period spanning from January 1, 2020, to July 31, 2023. State vaccination coverage was estimated as October 2, 2021. Pearson correlation coefficients reflecting significant relationships (p <

medRxiv preprint doi: https://doi.org/10.1101/2024.01.21.24301582; this version posted August 12, 2024. The copyright holder for this preprint (which was not certified by peer review) is the author/funder, who has granted medRxiv a license to display the preprint in perpetuity.
It is made available under a CC-BY 4.0 International license .

0.05) are shown above the scatter plots. (A) The dependent variable is excess mortality,not adjusetd for age. (B) The dependent variable is age-adjusted excess mortality.

medRxiv preprint doi: https://doi.org/10.1101/2024.01.21.24301582; this version posted August 12, 2024. The copyright holder for this preprint (which was not certified by peer review) is the author/funder, who has granted medRxiv a license to display the preprint in perpetuity.
It is made available under a CC-BY 4.0 International license .

Supplementary Figure 1.

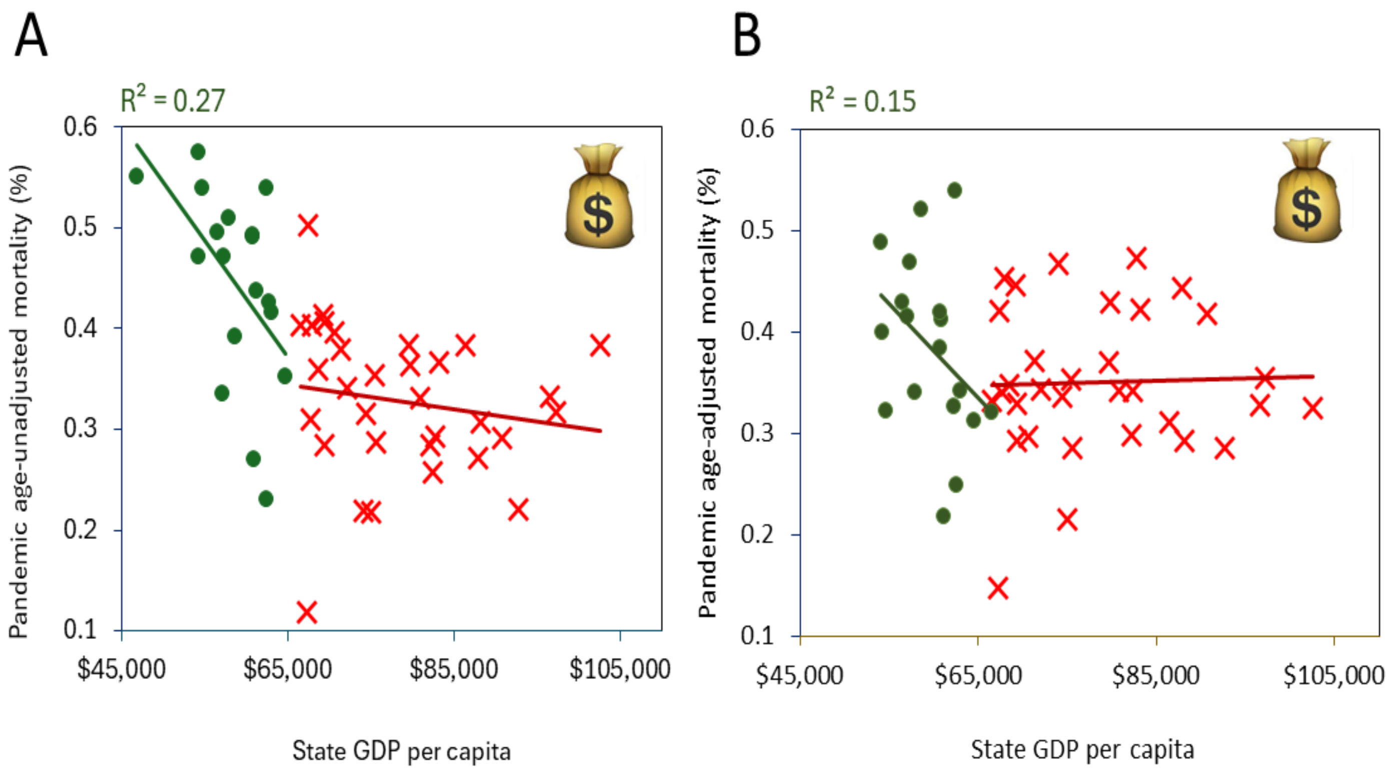

medRxiv preprint doi: https://doi.org/10.1101/2024.01.21.24301582; this version posted August 12, 2024. The copyright holder for this preprint (which was not certified by peer review) is the author/funder, who has granted medRxiv a license to display the preprint in perpetuity.
It is made available under a CC-BY 4.0 International license .

Supplementary Figure 2.

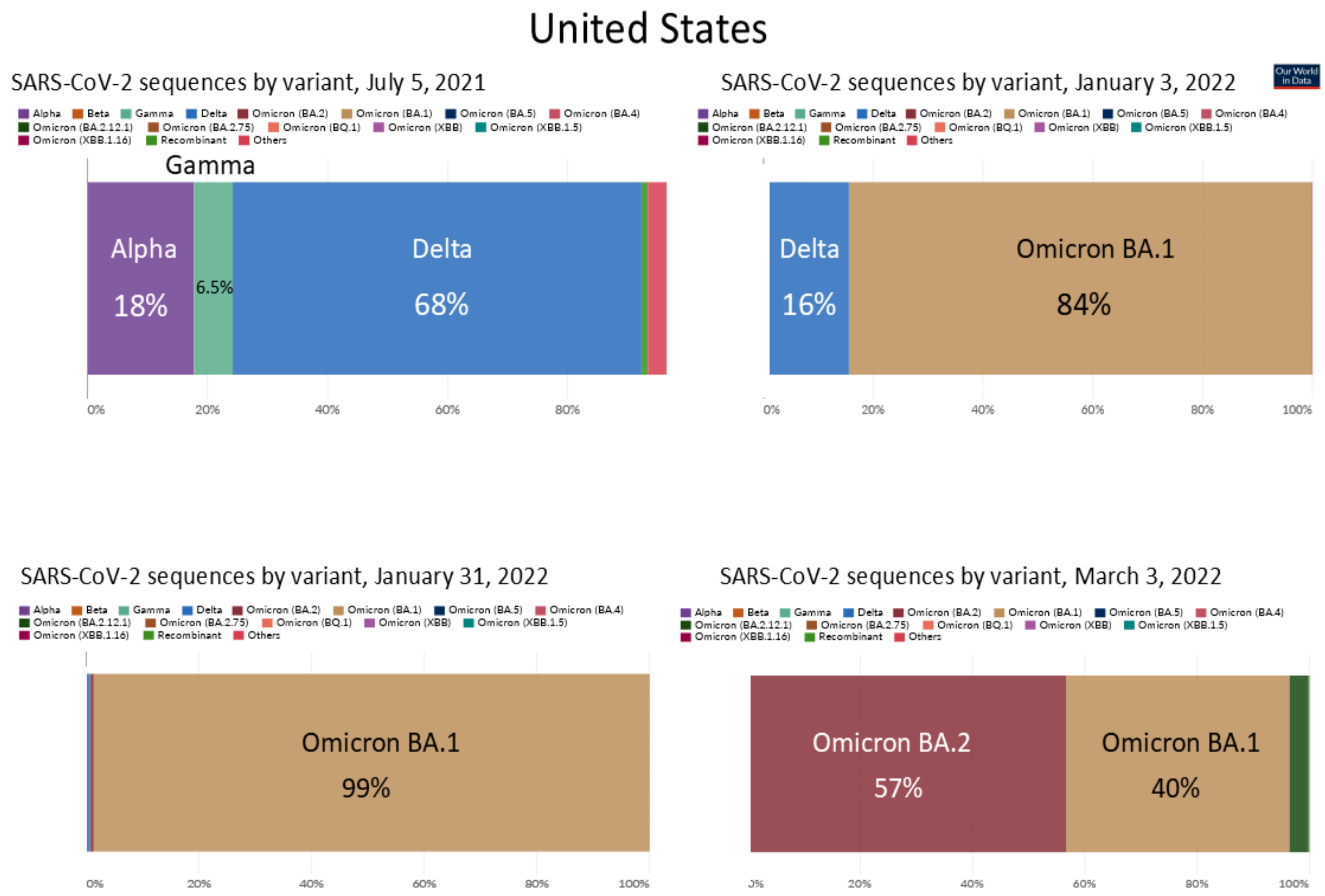

medRxiv preprint doi: https://doi.org/10.1101/2024.01.21.24301582; this version posted August 12, 2024. The copyright holder for this preprint (which was not certified by peer review) is the author/funder, who has granted medRxiv a license to display the preprint in perpetuity.
It is made available under a CC-BY 4.0 International license .

Supplementary Figure 3.

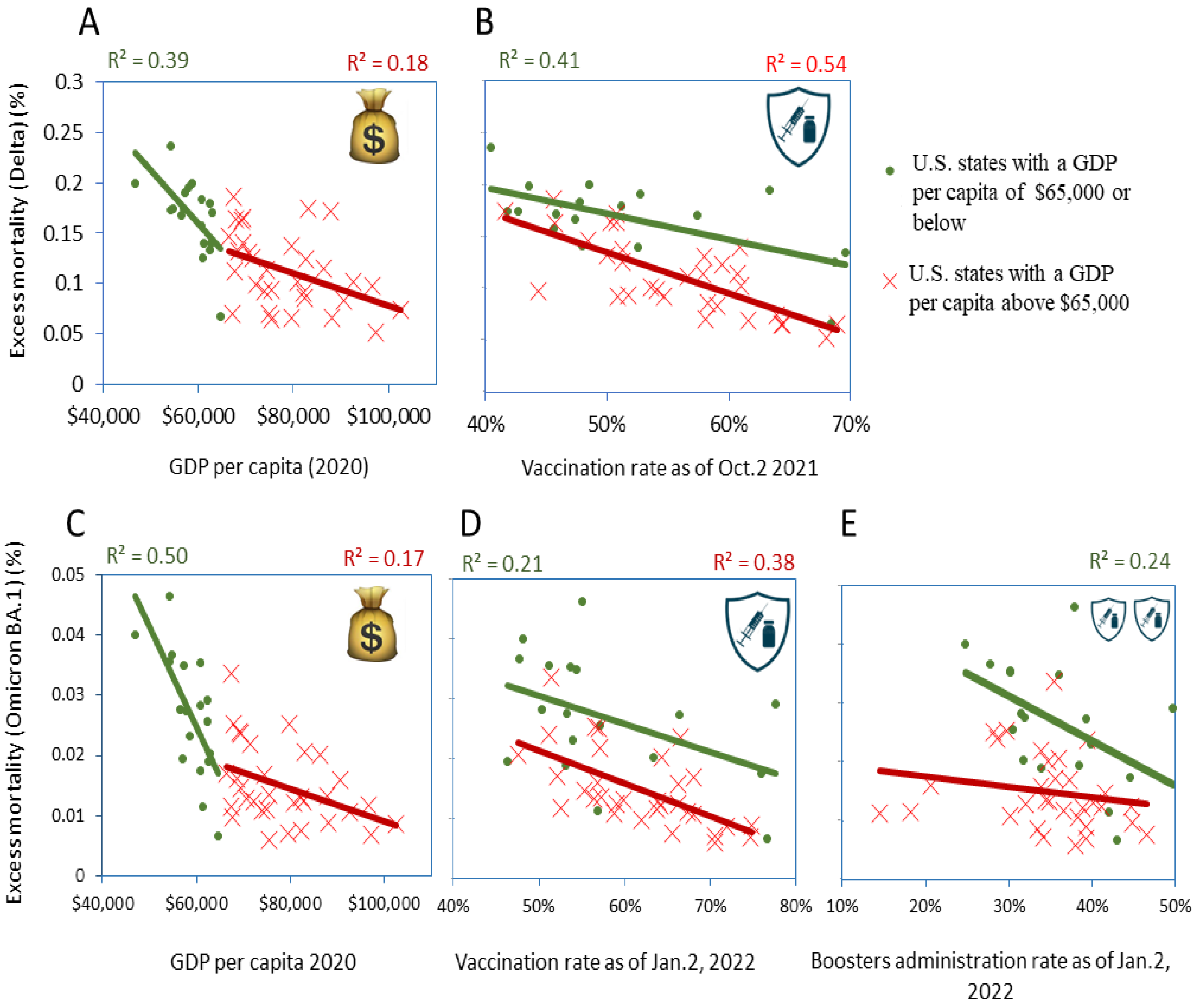

medRxiv preprint doi: https://doi.org/10.1101/2024.01.21.24301582; this version posted August 12, 2024. The copyright holder for this preprint (which was not certified by peer review) is the author/funder, who has granted medRxiv a license to display the preprint in perpetuity.
It is made available under a CC-BY 4.0 International license .

Supplementary Figure 4.

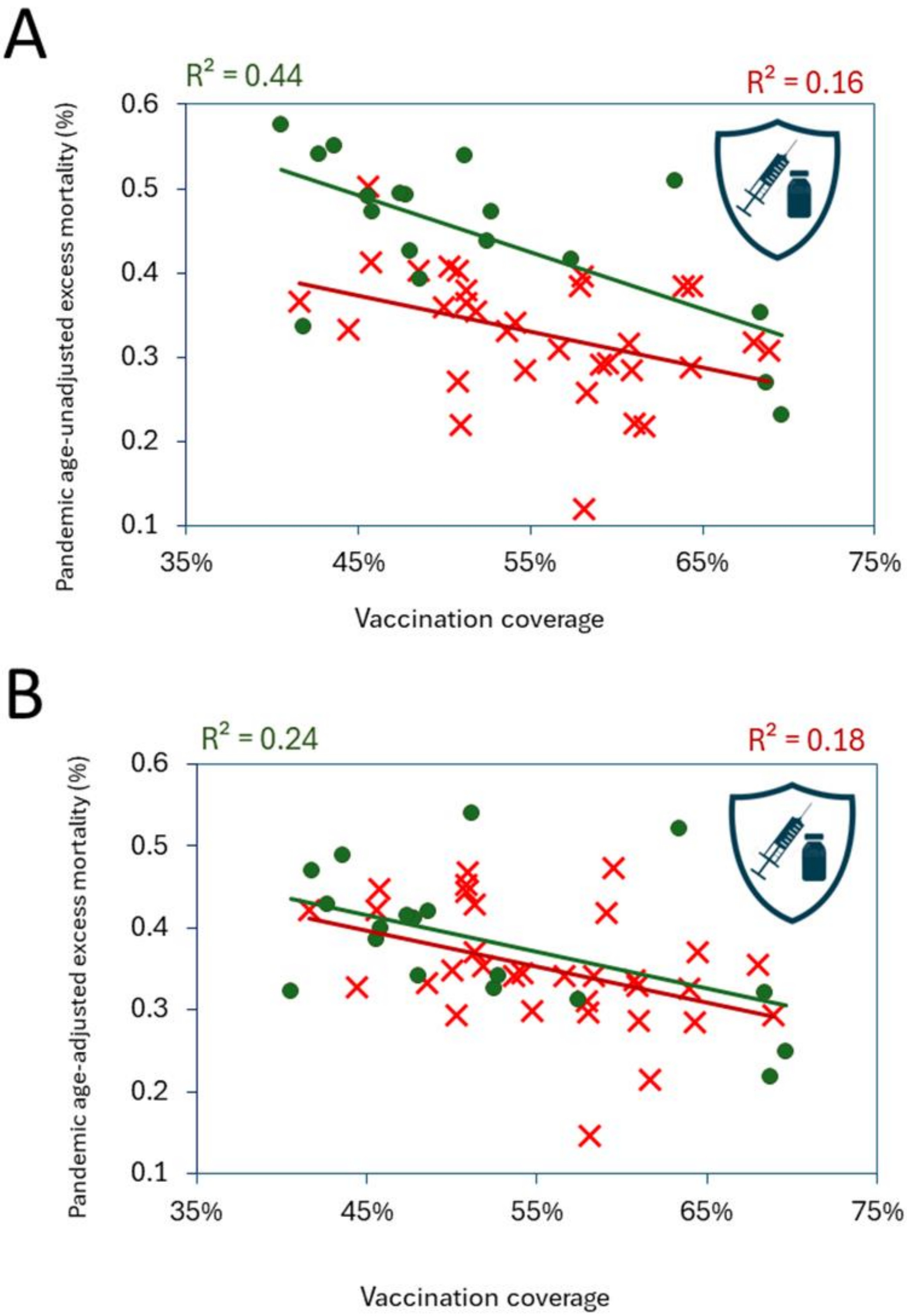

Supplement: Supplement 2 [file NIHPP2024.01.21.24301582v2-supplement-2.pdf]
