# Supplementary material for: Impact of Vaccination Rates and Gross Domestic Product on COVID-19 Pandemic Mortality Across United States
Source: medRxiv. 2024 Jan 22:2024.01.21.24301582. Preprint. [Version 1] doi: 10.1101/2024.01.21.24301582 (PMC10836123; doi:10.1101/2024.01.21.24301582)
Supplement: Supplementary file 1 [file media-1.docx]

|  | Time | Excess mortality (percentage from a state population) |  |  |  | Vaccination |  | Boosters | GDP per capita ($) |
| --- | --- | --- | --- | --- | --- | --- | --- | --- | --- |
|  |  |  |  |  |  |  |  |  |  |
|  | Year | 2020-2021 | 2021-2022 | 2022 | 2022-2023 | 2021 | 2022 | 2022 | 2020 |
|  | Month | Feb.1- 2020 - Jun.30, 2021 | July-1,2021 -Jan.- 31, 2022 | Feb.1 -Mar.31 | Apr.-Apr. | Oct.2 | Jan. 2 |  |  |
|  | State | pre- | Delta | Omicron | Post | % |  |  | Bureau of Economic Analysis |
|  |  | Delta |  | BA.1 | BA.1 |  |  |  |  |
| AL | Alabama | 0.296 | 0.176 | 0.037 | 0.067 | 42.7 | 47.7 | 27.7 | $54,652.00 |
| AK | Alaska | 0.106 | 0.176 | 0.013 | 0.075 | 50.8 | 56.4 | 34.8 | $87,782.00 |
| AZ | Arizona | 0.292 | 0.182 | 0.026 | 0.066 | 51.2 | 57.1 | 30.4 | $62,342.00 |
| AR | Arkansas | 0.252 | 0.175 | 0.036 | 0.071 | 45.8 | 51.2 | 30.2 | $54,141.00 |
| CA | California | 0.198 | 0.084 | 0.016 | 0.064 | 59.1 | 66.3 | 33.9 | $90,696.00 |
| CO | Colorado | 0.141 | 0.126 | 0.012 | 0.063 | 59.5 | 66.3 | 41.1 | $82,730.00 |
| CT | Connecticut | 0.202 | 0.066 | 0.009 | 0.047 | 68.9 | 74.8 | 39.3 | $88,185.00 |
| DE | Delaware | 0.213 | 0.117 | 0.02 | 0.08 | 57.9 | 64.3 | 34.7 | $86,413.00 |
| FL | Florida | 0.192 | 0.171 | 0.02 | 0.058 | 57.4 | 63.4 | 31.7 | $62,965.00 |
| GA | Georgia | 0.232 | 0.163 | 0.024 | 0.07 | 45.7 | 51.2 | 28.6 | $69,222.00 |
| HI | Hawaii | 0.04 | 0.069 | 0.012 | 0.064 | 58.1 | 63.7 | 18.2 | $67,299.00 |
| ID | Idaho | 0.13 | 0.176 | 0.019 | 0.071 | 41.8 | 46.3 | 38.4 | $57,072.00 |
| IL | Illinois | 0.205 | 0.102 | 0.012 | 0.049 | 53.6 | 64.2 | 38.8 | $80,812.00 |
| IN | Indiana | 0.208 | 0.146 | 0.017 | 0.045 | 48.5 | 52.1 | 35.6 | $66,483.00 |
| IA | Iowa | 0.167 | 0.099 | 0.012 | 0.044 | 54.1 | 59 | 44.7 | $72,065.00 |
| KS | Kansas | 0.179 | 0.126 | 0.022 | 0.055 | 51.3 | 57.1 | 33.8 | $71,326.00 |
| KY | Kentucky | 0.201 | 0.193 | 0.035 | 0.081 | 52.7 | 54.3 | 36 | $57,283.00 |
| LA | Louisiana | 0.295 | 0.158 | 0.028 | 0.042 | 45.6 | 50.4 | 31.4 | $60,780.00 |
| ME | Maine | 0.084 | 0.128 | 0.017 | 0.096 | 68.7 | 75.9 | 44.5 | $60,870.00 |
| MD | Maryland | 0.177 | 0.066 | 0.006 | 0.011 | 64.3 | 70.5 | 38 | $75,528.00 |
| MA | Massachusetts | 0.156 | 0.052 | 0.007 | 0.041 | 68 | 74.7 | 39.2 | $97,239.00 |
| MI | Michigan | 0.216 | 0.14 | 0.011 | 0.052 | 52.5 | 56.9 | 41.9 | $61,234.00 |
| MN | Minnesota | 0.119 | 0.087 | 0.008 | 0.035 | 58.3 | 65.5 | 46.5 | $82,344.00 |
| MS | Mississippi | 0.354 | 0.2 | 0.04 | 0.08 | 43.6 | 48.1 | 24.8 | $46,848.00 |
| MO | Missouri | 0.212 | 0.142 | 0.019 | 0.048 | 48 | 53.1 | 33.8 | $62,645.00 |
| MT | Montana | 0.185 | 0.204 | 0.023 | 0.077 | 48.6 | 54 | 39.8 | $58,656.00 |
| NE | Nebraska | 0.147 | 0.092 | 0.013 | 0.048 | 54.7 | 59.9 | 40.1 | $82,149.00 |
| NV | Nevada | 0.2 | 0.165 | 0.025 | 0.049 | 50.8 | 56.6 | 29.8 | $67,895.00 |
| NH | New Hampshire | 0.087 | 0.07 | 0.011 | 0.069 | 61.6 | 67.3 | 14.5 | $74,998.00 |
| NJ | New Jersey | 0.267 | 0.066 | 0.007 | 0.02 | 64.4 | 70.6 | 34.2 | $79,594.00 |
| NM | New Mexico | 0.259 | 0.198 | 0.027 | 0.068 | 63.4 | 66.4 | 39.3 | $57,852.00 |
| NY | New York | 0.289 | 0.074 | 0.009 | 0.042 | 63.9 | 71.9 | 33.5 | $102,587.00 |
| NC | North Carolina | 0.18 | 0.132 | 0.016 | 0.069 | 50 | 56.9 | 20.6 | $68,539.00 |
| ND | North Dakota | 0.206 | 0.099 | 0.012 | 0.037 | 44.4 | 52.6 | 36.6 | $96,570.00 |
| OH | Ohio | 0.197 | 0.164 | 0.015 | 0.027 | 50.3 | 55.3 | 41.5 | $69,408.00 |
| OK | Oklahoma | 0.231 | 0.184 | 0.035 | 0.076 | 47.8 | 53.6 | 30.1 | $60,788.00 |
| OR | Oregon | 0.106 | 0.146 | 0.024 | 0.113 | 60.9 | 66.5 | 39.4 | $69,318.00 |
| PA | Pennsylvania | 0.208 | 0.131 | 0.013 | 0.037 | 58 | 63.9 | 32 | $70,612.00 |
| RI | Rhode Island | 0.199 | 0.067 | 0.007 | 0.03 | 68.4 | 76.7 | 43 | $64,620.00 |
| SC | South Carolina | 0.268 | 0.169 | 0.028 | 0.073 | 47.4 | 53.2 | 31.8 | $56,441.00 |
| SD | South Dakota | 0.226 | 0.095 | 0.014 | 0.035 | 51.8 | 57.2 | 34.4 | $75,406.00 |
| TN | Tennessee | 0.238 | 0.186 | 0.034 | 0.052 | 45.6 | 51.4 | 35.4 | $67,374.00 |
| TX | Texas | 0.232 | 0.139 | 0.025 | 0.054 | 51.3 | 57 | 28.1 | $79,736.00 |
| UT | Utah | 0.096 | 0.093 | 0.011 | 0.038 | 50.9 | 58.8 | 30.1 | $73,995.00 |
| VT | Vermont | 0.129 | 0.134 | 0.022 | 0.174 | 69.6 | 77.6 | 49.7 | $62,401.00 |
| VA | Virginia | 0.154 | 0.115 | 0.017 | 0.059 | 60.7 | 68.1 | 37.3 | $74,375.00 |
| WA | Washington | 0.076 | 0.108 | 0.011 | 0.077 | 61 | 68 | 38.3 | $92,703.00 |
| WV | West Virginia | 0.232 | 0.238 | 0.046 | 0.115 | 40.5 | 55.1 | 37.8 | $54,278.00 |
| WI | Wisconsin | 0.148 | 0.112 | 0.01 | 0.039 | 56.6 | 62 | 44.8 | $67,659.00 |
| WY | Wyoming | 0.199 | 0.177 | 0.021 | 0.061 | 41.6 | 47.6 | 36 | $83,121.00 |
| PR | Puerto Rico | 0.121 | 0.08 | 0.008 | 0.131 | 70.7 | 77.1 | 28.9 | $32,641.00 |
| The table presents excess mortality and vaccination as a percentage of each state's population. |  |  |  |  |  |  |  |  |  |
